# Manganese homeostasis modulates fungal virulence and stress tolerance in *Candida albicans*

**DOI:** 10.1128/msphere.00804-23

**Published:** 2024-02-21

**Authors:** Manon Henry, Inès Khemiri, Faiza Tebbji, Rasmi Abu-Helu, Antony T. Vincent, Adnane Sellam

**Affiliations:** 1 Montreal Heart Institute/Institut de Cardiologie de Montréal, Université de Montréal, Montréal, Québec, Canada; 2 Department of Microbiology, Infectious Diseases and Immunology, Faculty of Medicine, Université de Montréal, Montréal, Québec, Canada; 3 Department of Medical Laboratory Sciences, Faculty of Health Professions, Al-Quds University, Jerusalem, Palestine; 4 Department of Animal Sciences, Université Laval, Quebec City, Québec, Canada; University of Georgia, USA

**Keywords:** *Candida albicans*, manganese homeostasis, unfolded protein response, antifungal stress, fungal virulence

## Abstract

Due to the scarcity of transition metals within the human host, fungal pathogens have evolved sophisticated mechanisms to uptake and utilize these micronutrients at the infection interface. While considerable attention was turned to iron and copper acquisition mechanisms and their importance in fungal fitness, less was done regarding either the role of manganese (Mn) in infectious processes or the cellular mechanism by which fungal cells achieve their Mn-homeostasis. Here, we undertook transcriptional profiling in the pathogenic fungus *Candida albicans* experiencing both Mn starvation and excess to capture biological processes that are modulated by this metal. We uncovered that Mn scarcity influences diverse processes associated with fungal fitness including invasion of host cells and antifungal sensitivity. We show that Mn levels influence the abundance of iron and zinc emphasizing the complex crosstalk between metals. The deletion of *SMF12*, a member of Mn Nramp transporters, confirmed its contribution to Mn uptake. *smf12* was unable to form hyphae and damage host cells and exhibited sensitivity to azoles. We found that the unfolded protein response (UPR), likely activated by decreased glycosylation under Mn limitation, was required to recover growth when cells were shifted from an Mn-starved to an Mn-repleted medium. RNA-seq profiling of cells exposed to Mn excess revealed that UPR was also activated. Furthermore, the UPR signaling axis Ire1-Hac1 was required to bypass Mn toxicity. Collectively, this study underscores the importance of Mn homeostasis in fungal virulence and comprehensively provides a portrait of biological functions that are modulated by Mn in a fungal pathogen.

**IMPORTANCE:**

Transition metals such as manganese provide considerable functionality across biological systems as they are used as cofactors for many catalytic enzymes. The availability of manganese is very limited inside the human body. Consequently, pathogenic microbes have evolved sophisticated mechanisms to uptake this micronutrient inside the human host to sustain their growth and cause infections. Here, we undertook a comprehensive approach to understand how manganese availability impacts the biology of the prevalent fungal pathogen, *Candida albicans*. We uncovered that manganese homeostasis in this pathogen modulates different biological processes that are essential for host infection which underscores the value of targeting fungal manganese homeostasis for potential antifungal therapeutics development.

## INTRODUCTION

Transition metals, such as iron (Fe), copper (Cu), zinc (Zn), and manganese (Mn), provide considerable functionality across biological systems as they are used as cofactors for catalytic enzymes and are thought to be required for the activity of one-third of a cellular proteome (1). Metal ions alter the physicochemical properties of proteins, which promotes enzymatic catalysis, stabilization of protein structure, and electron and chemical group transfers (2). At elevated concentrations, trace metals exhibit high toxicity which imposes a tight regulation of their abundance at homeostatic levels. Trace metals are also deterministic for host-pathogen interaction as they are sequestered by the host to limit the proliferation of microbial pathogens, a process known as nutritional immunity (2, 3). This process is achieved by the production of sequestering molecules such as calprotectin and siderocalin that chelate Mn, Zn, and Fe (4–6). Furthermore, ion metals are also sequestered in storage tissues and intracellular organelles which also limits the availability of those nutrients for uptake and use by invading pathogens (2, 5). Inversely, during infection, some immune cells tackle bacterial pathogens by releasing toxic levels of Cu or Zn which promotes microbial killing and attenuation of infectivity (2).

As transition metal availability is very limited inside the human host, fungal pathogens have evolved sophisticated mechanisms to uptake and utilize these micronutrients at the infection interface. For instance, Fe acquisition by heme utilization and siderophore-mediated uptake is recognized as key virulence factors in many important fungal human pathogens including *Candida albicans*, *Aspergillus fumigatus*, and *Cryptococcus neoformans* (7–10). Similarly, Cu uptake is tightly controlled by the transcription factor Mac1 which is essential for fungal fitness *in vivo* (11–13). Zn internalization by Zrts transporters or by the Zn scavenger Pra1 in *C. albicans* was also shown to be critical for the infectivity of this yeast and the expression of virulence traits (14–16). While considerable attention was turned to Fe, Cu, and Zn acquisition mechanisms and their importance in fungal fitness, the role of Mn in infectious processes or the cellular mechanism by which fungal cells achieve their Mn-homeostasis is not well understood.

Mn is an essential trace element throughout the tree of life and serves as a cofactor for many enzymes such as polymerases, glycosyltransferases, and superoxide dismutases (17). Mammalian hosts restrict Mn availability by the Mn-chelating protein, calprotectin which contributes to antibacterial and antifungal defenses (18–20). So far, the counteracting mechanisms to Mn sequestration by the host allowing Mn uptake and utilization by fungal pathogens remain unknown. In the budding yeast *Saccharomyces cerevisiae*, Mn uptake is achieved by Smf1 and Smf2 transporters of the NRAMP (natural resistance-associated macrophage protein) family in addition to the phosphate permease Pho84 which is also a low-affinity Mn transporter (21–25). Unlike Fe, Zn, and Cu transport that are subject to transcriptional control by metal-sensing transcription factors, Mn transport in *S. cerevisiae* is regulated at the post-translational level by ubiquitination and through changes in subcellular localization of Smf transporters (21, 22, 25). Under Mn sufficiency, Smf2 and Smf1 transit into the Golgi and are ubiquitinated by the E3 ubiquitin ligase Rsp5 which directs them to the vacuole for subsequent degradation, a process facilitated by adaptor proteins including Bsd2, Tre1, and Tre2 (21). When Mn is depleted, Smf1 stability increases through conformation changes, which make it not recognizable by Rsp5/Bsd2 and subsequently localize to the plasma membrane for Mn uptake (22). While the mechanisms preserving Mn homeostasis are well known in the saprophytic yeast *S. cerevisiae*, they remain largely unexplored in the rest of fungal species including human fungal pathogens. In *C. albicans*, deletion of *PHO84* had no impact on Mn uptake suggesting a diverging role for this transporter as compared to the budding yeast (26). Mn was also shown to be a critical cofactor for the *C. albicans* Mn-superoxide dismutases Sod2 and Sod3 and their contribution to oxidative stress defense (27–29).

In the current study, we undertook transcriptional profiling of *C. albicans* cells experiencing both Mn starvation and excess to comprehensively capture biological processes that are modulated by Mn abundance. We uncovered that Mn scarcity influences diverse biological processes associated with fungal virulence including morphogenetic switch, metabolic flexibility, invasion of different host cells, and antifungal and unfolded protein response (UPR) stress responses. We also deleted one of the four Nramp transporters in *C. albicans*, named *SMF12* (Orf19.2270), and confirmed its contribution to Mn uptake and fungal fitness *in vivo*. While preparing this manuscript for submission, Wildeman et al. (27) published a similar work confirming the contribution of Smf12 to Mn homeostasis and its requirement for Mn-superoxide dismutase activity and oxidative stress resistance. We also analyzed the transcriptome of *C. albicans* cells exposed to Mn excess and uncovered that the UPR signaling was activated. Accordingly, we found that Ire1, the master regulator of UPR signaling in fungi, was essential to bypass Mn toxicity. Our RNA-seq analysis of the Mn-modulated transcriptome in *C. albicans* provides a global framework for future studies to examine the link between Mn metabolism and essential functions that modulate fungal virulence and ﬁtness inside the host.

## RESULTS AND DISCUSSION

### Modulation of Mn content perturbs fungal growth

**Fig 1:**
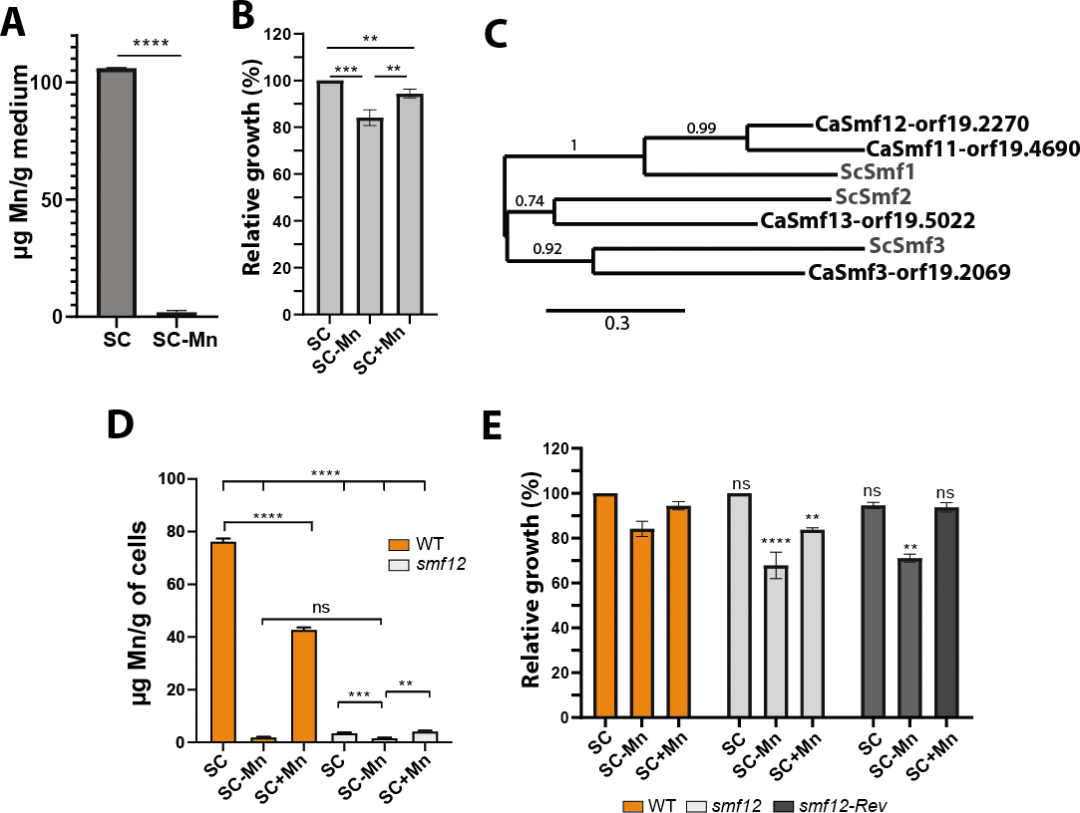
Modulation of Mn content perturbs fungal growth. (**A**) Mn quantification by ICP-MS in conventional SC and Mn-free SC media (SC-Mn). Mn levels were determined by analyzing powders of the conventional yeast nitrogen base (YNB) and the Mn-free YNB used to prepare the SC and the SC-Mn media, respectively. (**B**) Impact of Mn depletion on *C. albicans* growth. WT strain (SC5314) was grown at 30°C for 13 h in SC-Mn and SC + Mn media. Growth was normalized to the SC condition. (**C**) Phylogram of Smf transporters in *C. albicans* (CaSmf11-orf19.4690, CaSmf12-orf19.2270, CaSmf13-orf19.5022, and CaSmf3-orf19.2069) and *S. cerevisiae* (ScSmf1, ScSmf2, and ScSmf3). The multiple sequence alignment for the four Smf orthologs and the phylogenetic tree was generated using Clustal Omega. The branch length is proportional to the number of substitutions per site. (**D**) Mn uptake in the *smf12* mutant. Mn levels were determined by ICP-MS in both exponentially grown WT (SN148-CIp20) and *smf12* strains in SC, SC-Mn, and SC + Mn media at 30°C. (**E**) Impact of Mn depletion on *smf12* mutant. WT strain (SN148-CIp20), *smf12,* and the complemented *smf12* (s*mf12*-Rev) strains were grown at 30°C for 13 h in SC-Mn or SC + Mn. Growth was normalized to the SC condition. Statistics are a two-way analysis of variance (ANOVA) with **** *P*-value < 0.00001, *** *P*-value < 0.0001, and ** *P*-value < 0.001; ns: nonsignificant.

Due to the absence of a specific Mn chelator, we used a commercial Mn-free synthetic complete growth medium (SC-Mn) to assess the impact of Mn limitation on the growth of *C. albicans*. Quantification of Mn levels using inductively coupled plasma-mass spectrometry (ICP-MS) confirmed the absence of Mn in the SC-Mn medium while showing the expected Mn amount in the conventional SC medium (SC; Fig. 1A). In the absence of Mn (SC-Mn), *C. albicans* exhibited a moderate growth reduction of roughly 20% as compared to the Mn replete condition (SC; Fig. 1B). Supplementation of SC-Mn medium with 1 mM MnCl_2_ (SC + Mn) restored the growth of *C. albicans* to a level comparable to that observed in SC medium. To further modulate the intracellular levels of Mn, we created a mutant of one of the four predicted Mn transporters, Smf12 (orf19.2270; Fig. 1C). The ICP-MS analysis confirmed the low level of internalized Mn in *smf12* mutant under either Mn depletion (SC-Mn) or repletion conditions (SC + Mn or SC) suggesting that Smf12 is a major transporter of Mn in *C. albicans* (Fig. 1D). Accordingly, *smf12* exhibited a significant growth defect in either Mn-repleted or depleted media as compared to the Wild type (WT) strain (Fig. 1E). Meanwhile, in the SC + Mn condition, a small but significant accumulation of Mn in the *smf12* mutant was perceived suggesting that Mn is being internalized by either other Smf transporters (27) or through passive diffusion (30). The moderate impact of *smf12* mutation on *C. albicans* growth in SC-Mn is likely attributed to the nonessentiality of Mn or the possibility that cells possess intracellular Mn reserves at trace levels that are mobilized under Mn deprivation. Taken together, these data suggest that modulation of intracellular Mn in *C. albicans* impacts the growth of this opportunistic yeast.

### Genome-wide transcriptional response of *C. albicans* to Mn starvation

**Fig 2:**
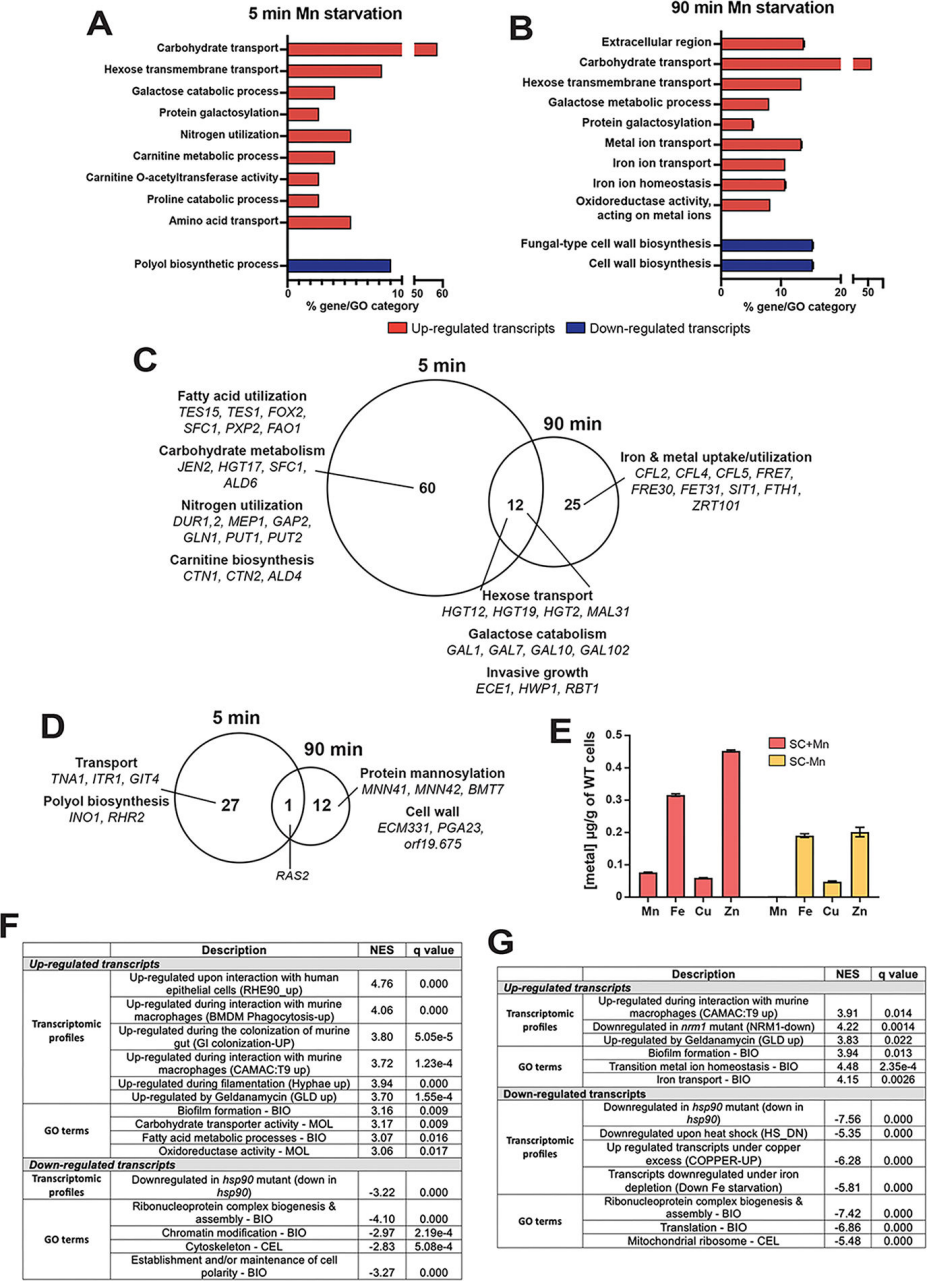
Transcriptomic analysis of *C. albicans* response to Mn starvation. (**A and B**) GO enrichment of upregulated and downregulated transcripts of *C. albicans* cells growing under Mn limitation compared to cells growing under Mn repletion at 5 (**A**) and 90 min (**B**). (**C and D**) Venn diagram showing overlaps between transcripts upregulated (**C**) or downregulated (**D**) under Mn depletion at 5 and 90 min. Relevant biological functions and their associated genes are shown. (**E**) Quantification by ICP-MS of Fe, Mn, Zn, and Cu in exponentially grown WT cells in SC-Mn and SC + Mn media at 30°C. (**F and G**) Gene set enrichment analysis (GSEA) of the *C. albicans* Mn-modulated transcriptome at 5 (**F**) and 90 min (**G**). The vertical black lines indicate the position of each of the genes modulated by Mn in the three ordered data sets (GLD_UP: transcripts upregulated by geldanamycin; Down in HSP90: transcripts downregulated in the repressible mutant Tet-HSP90, and HS_DN: transcripts downregulated by heat). The green curve represents the NES (normalized enrichment score) curve, which is the running sum of the weighted enrichment score obtained from GSEA software. NES and nominal *q*-value obtained from the GSEA are shown at the bottom of each plot. The complete GSEA correlations are listed in Table S2. False-Discovery Rate (*q*-value) of 1%.

Although Mn is an important metal for different microorganisms, the overall impact of its limitation on fungal biology is not well characterized so far. To define the cellular processes that are modulated by Mn in the pathogenic yeast *C. albicans*, we monitored the global gene expression dynamic under Mn insufficiency using RNA-seq. The transcriptome of cells growing in SC-Mn was compared to that of cells thriving in SC + Mn at 5 and 90 min to capture adaptive mechanisms of Mn starvation. Gene ontology (GO) analysis revealed that Mn limitation at 5 and 90 min induces significant metabolic reprogramming as reflected by the upregulation of transcripts related to carbohydrate transport, galactolysis, carnitine biosynthesis, and, fatty acid and nitrogen utilization (Fig. 2A and B; Table S1). The transcriptional activation of metabolic genes might reflect a compensatory response by *C. albicans* cells to sustain their metabolic needs as Mn is required for many metabolic metalloenzymes such as transferases, dehydrogenases, and oxidases (31). The 90-min time point was notable for the upregulation of genes involved in Fe transport (*FRE7*, *FRE30*, *CFL2,4,5*, *FTH1*, *RBT5*, and *SIT1*) and the downregulation of transcripts of mannosyltransferases (*MNN41*, *MNN42*, and *BMT7*) and cell wall integrity proteins (*ECM331* and *PGA23*; Fig. 2C through D). Genes modulated by Mn include different virulence factors required for the yeast-to-hyphae transition (*HWP1*) and the production of the cytolytic toxin Candidalysin (*ECE1*; Fig. 2C). Coactivation of morphogenesis genes in *C. albicans* together with Fe uptake and utilization was previously observed and might reflect the adaptive prediction phenomenon (32–34). This concept suggests that as fungal cells invade host cells, metabolic processes, such as metal uptake and utilization, are anticipatedly activated to accommodate the metabolic demands of fungal cells. Alternatively, the upregulation of Fe regulon might reflect a situation where this essential metal is depleted as a consequence of Mn starvation. Indeed, ICP-MS quantification showed that the abundance of Fe was significantly reduced in *C. albicans* cells under Mn limitation as compared to Mn-replete conditions (Fig. 2E). Thus, Mn depletion led to Fe deficiency in *C. albicans* as was observed in bacteria, plants, and algae (35–38). Furthermore, our ICP-MS data also show that Zn levels, but not Cu, were reduced which might also explain the upregulation of the Zn transporter Zrt101 at 90 min growth in SC-Mn (Fig. 2C; Table S1). These results indicate that *C. albicans* Fe and Zn homeostasis are modulated by intracellular Mn levels.

To further explore the biological processes modulated by Mn abundance, we used gene set enrichment analysis (GSEA) to elucidate resemblance with the set of the previously published *C. albicans* transcriptional profiling experiments (39). Upregulated transcripts under Mn starvation exhibit significant similarity with transcriptional profiles reflecting different contexts of host-*C. albicans* interactions, including the colonization of the mammalian gut and infection of human oral epithelial cells and macrophages (Fig. 2F through G; Table S2). This finding suggests that Mn depletion is a situation that *C. albicans* might encounter *in vivo*. Furthermore, transcriptional programs associated with invasive filamentous growth and biofilm formation were also positively correlated with activated transcripts in Mn-starved cells. Intriguingly, GSEA revealed that both 5- and 90-min Mn starvation transcriptomes were correlated with gene signatures of cells where the essential chaperone Hsp90 was inhibited (cells treated with geldanamycin, transcripts downregulated in *hsp90* mutant, and transcripts repressed by heat; Fig. 2F through G; Table S2). Together, these results imply that Mn limitation generates a transcriptional signature that is reminiscent of heat stress and the expression of fungal virulence.

### Mn starvation induces the unfolded protein response

**Fig 3:**
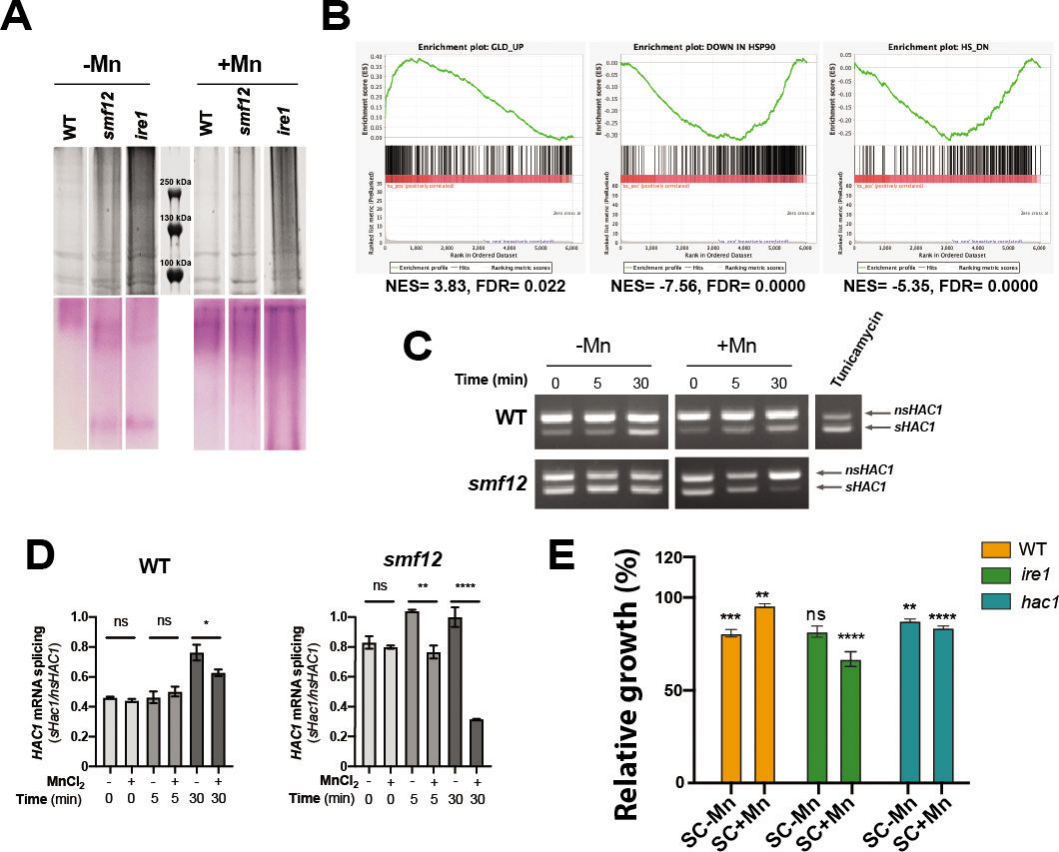
Mn limitation promotes the UPR in *C. albicans.* (**A**) Impact of *C. albicans* Mn homeostasis on protein glycosylation. *C. albicans* cells were grown for the RNA-seq experiment, and proteins were analyzed on SDS-PAGE with silver stain (upper panel). The same samples on SDS-PAGE were stained using the Glycoprotein Staining Kit (lower panel). (**B**) GSEA graphs of significant correlations between the Mn-depletion transcriptome and Hsp90 inhibition. (**C and D**) Mn starvation induces *HAC1* mRNA splicing. WT (SN148-CIp20) and *smf12* cells were grown in SC-Mn at the indicated time, and *HAC1* splicing was assessed using RT-PCR (**C**). The glycosylation inhibitor and the UPR inducer tunicamycin were used as a positive control. *nsHAC1*, nonspliced *HAC1; sHAC1*, spliced *HAC1*. (**D**) Quantitative assessment of *HAC1* splicing. The intensity of PCR bands was measured using ImageJ, and data are presented as the ratio of the sHAC1/nsHAC1 PCR band intensity. (**E**) Impact of Mn depletion on the growth of *ire1* and *hac1* mutants. *C. albicans* strains were grown at 30°C for 13 h in SC-Mn or SC + Mn. Growth was normalized to the SC condition. Statistics are ANOVA test with **** *P*-value < 0.00001, *** *P*-value < 0.0001, and ** *P*-value < 0.001; ns: nonsignificant.

Transcript levels of three mannosyltransferases (*MNN41*, *MNN42*, and *BMT7*) which are enzymes required for the glycosylation of proteins, a process required for protein secretion and cell wall integrity (40), were reduced under Mn limiting conditions (Fig. 2D). Given the requirement of Mn for the activity of these enzymes, this might reflect a reduced mannosyltransferases activity in *C. albicans* cells. First, we tested the impact of Mn abundance on the glycosylation levels of *C. albicans* proteins in the WT strain under both Mn limitation and repletion. Surprisingly, under Mn scarcity, we noticed staining of glycosylated proteins with high molecular weight (>250 kDa) as compared to Mn repletion exhibiting staining of proteins with molecular weight higher than 130 kDa (Fig. 3A). Under Mn limitation, while the WT strain displayed staining of glycosylated proteins with exclusively high molecular weight (>250 kDa), the *smf12* mutant exhibited staining of proteins with lower molecular weight (<100 kDa) that is indicative of a decrease in the amount and the length of glycosyl residues linked to proteins (Fig. 3A). This observed that weight shift is related to a shortage of intracellular Mn as repletion led to a profile indistinguishable from that of the WT strain. Furthermore, our RNA-seq analysis of *C. albicans* cells experiencing Mn starvation revealed a transcriptional signature similar to that of cells where Hsp90 is either genetically or pharmacologically compromised (Fig. 3B). As Hsp90 primary role is to ensure correct folding and stability of proteins, we hypothesized that upon Mn depletion, this functionality is requested as a part of the UPR triggered by a decrease of protein glycosylation as previously reported (40). In eukaryotic cells, UPR is a signaling pathway activated by multiple endoplasmic reticulum (ER) stresses to preserve protein homeostasis (41). ER stress is sensed by the Ire1, an ER-located transmembrane endoribonuclease that excises an unconventional intron from the transcription factor *HAC1* mRNA which leads to its activation and induction of the UPR transcriptional response (42). To test whether Mn starvation activates UPR response in *C. albicans*, we used reverse transcription-PCR (RT-PCR) to assess the splicing variants of *HAC1* as a proxy of Ire1 activity in cells exposed to Mn starvation at different time points (0, 5, and 30 min). WT cells growing for 30 min under Mn limitation exhibited an increased level of the spliced form of *HAC1* as compared to the repleted condition (Fig. 3C through D). Splicing of *HAC1* was more marked in *smf12* mutant in the absence of Mn and almost entirely turned off at 30 min of growth in the repleted medium. We also found that *ire1* mutant exhibited a glycosylation pattern similar to that of *smf12* when Mn was omitted from the growth medium; however, *ire1* was insensitive to Mn supplementation as the glycosylation pattern remained unchanged (Fig. 3A). These data suggest that Mn starvation induces UPR response that is signaled by the Ire1-Hac1 regulatory axis. Accordingly, growth alleviation by Mn supplementation observed in WT strain under Mn starvation was not perceived in either *ire1* or *hac1* mutants (Fig. 3E). Recently, the UPR signaling pathway was also shown to be essential for Fe uptake in *C. albicans* by controlling localization of the high-affinity Fe permease Ftr1 to the cell membrane (43). Thus, UPR signaling appears to play an important role for both Fe and Mn homeostasis in *C. albicans*. Intriguingly, in addition to *ire1* not being rescued by Mn supplementation, it also manifested a growth defect under these conditions when compared to the Mn-starved medium (Fig. 3E). This can be attributed to the possibility that the 1 mM of Mn utilized might be toxic to *ire1*.

### Mn modulates host invasion and interaction with immune cells

**Fig 4:**
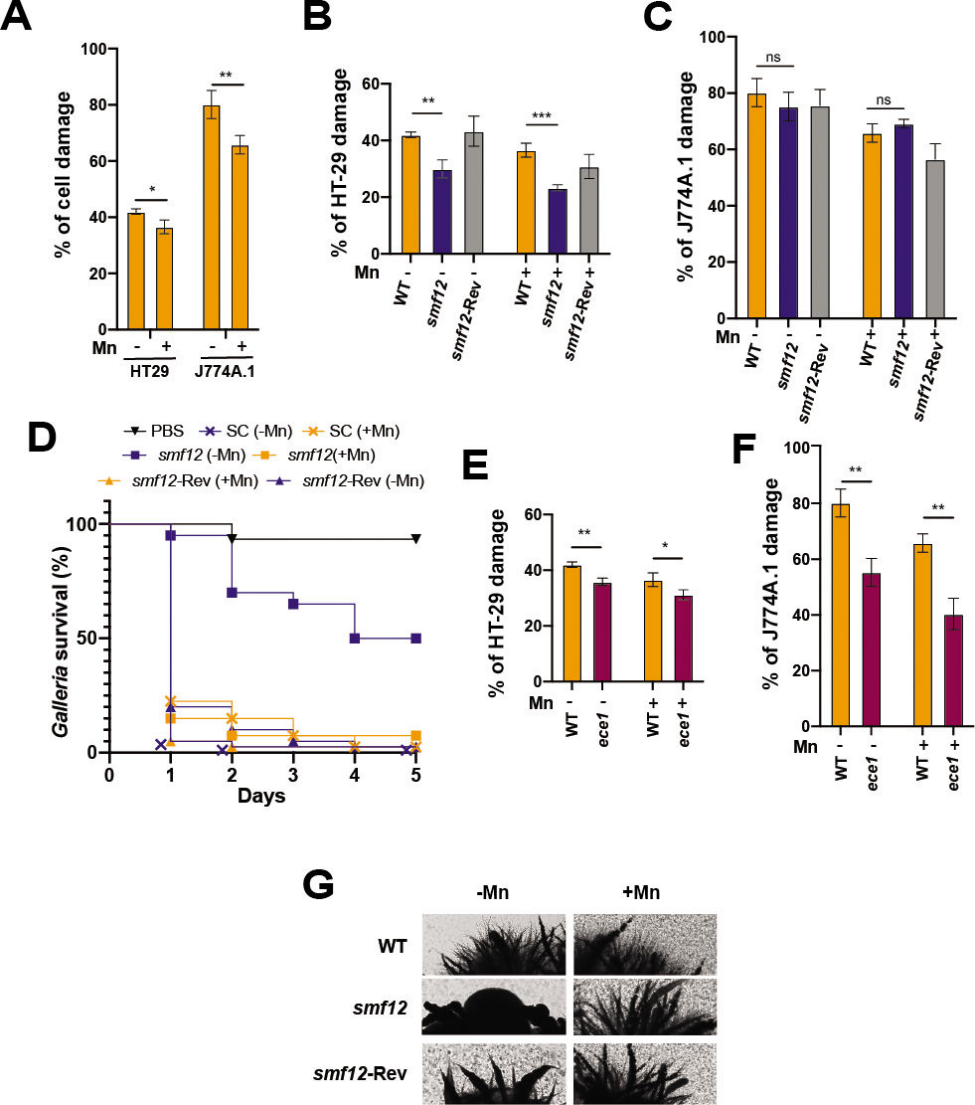
Mn abundance modulates fungal virulence (**A–C**) The effect of Mn abundance in preculture medium on *C. albicans* WT (SN148-CIp20) (**A**) and *smf12* infectivity of HT-29 human enterocytes (**B**) and J774A.1 murine macrophages (**C**). Cell damage was assessed using the lactate dehydrogenase (LDH) release assay and was calculated as a percentage of LDH activity as described in the method section. (**D**) Impact of Mn levels in *C. albicans* preculture medium and *smf12* mutation on *G. mellonella* larvae infection. WT (SN148-CIp20), *smf12,* and *smf12* complemented (*smf12*-Rev) strains, together with the PBS (Phosphate buffered saline) control, were injected into *G. mellonella* larvae, and survival was monitored daily for 5 days. (**E–F**) Effect of Mn levels on the expression of candidalysin. WT (SN250) and *ece1* strains precultured in either Mn-depleted or repleted growth media were tested for their ability to cause damage to HT-29 enterocytes and J774A.1 macrophages. Statistics are ANOVA test with *** *P*-value < 0.0001, ** *P*-value < 0.001, and * *P*-value < 0.05; ns: nonsignificant. (**G**) *smf12* morphogenetic defect. Colony morphologies of the WT (SN148-CIp20), *smf12,* and the complemented strain (*smf12*-Rev) after 3 days at 37°C on solid Spider medium under both Mn scarcity and repletion.

As the Mn-starvation transcriptome was similar to that expressed during *C. albicans* interaction with different host cells, we wanted to test the impact of Mn homeostasis on the ability of this yeast to damage the host. We found that *C. albicans* cells pregrown in Mn-starved medium cause more damage to both human enterocytes and murine macrophages than those thriving in Mn-repleted medium (Fig. 4A). The ability of *smf12* mutant to damage HT-29 enterocytes was significantly impaired as compared to the parental WT or the complemented strains regardless of Mn levels in fungal precultures (Fig. 4B). However, when coincubated with the J774.A.1 murine macrophage, *smf12* mutant exhibited a similar damage level as the WT strain (Fig. 4C). We also used the *Galleria mellonella* larvae-*C. albicans* model of systemic candidiasis to assess the impact of Mn levels in fungal precultures on the infectivity of both WT and *smf12* strains. On the first day of infection, *C. albicans* WT pregrown under Mn sufficiency resulted in the death of 80% of *Galleria* larvae, whereas those cultured in Mn-depleted medium caused 95% death (Fig. 4D). While *smf12* mutant pregrown under Mn replete condition exhibited a similar infectivity rate as the WT strain, *smf12* precultured under Mn limiting condition exhibited an attenuated virulence with a mortality rate of 5% and 50% at days 1 and 5, respectively, as compared to WT strain that led to 100% mortality at day 1 (Fig. 4D). Together, these findings suggest that Mn starvation promotes fungal invasiveness as was shown for other essential metals, and Smf12-mediated Mn homeostasis contributes to fungal virulence.

As the *ECE1* gene encoding candidalysin was upregulated under Mn limitation, we assessed whether the enhanced damage to host cells under this condition was mediated by the activity of this fungal cytolytic toxin. Consistent with previous work, *ece1* mutant caused less damage as compared to the WT strain (44); however, the enhanced damage reported under Mn limitation was also perceived in *ece1* (Fig. 4E through F). This suggests that Mn modulation of fungal invasiveness is independent of the activity of candidalysin. We did not notice any significant difference regarding the ability of *C. albicans* WT strain to form filaments when using a hyphae-promoting growth medium with depleted and repleted Mn levels (Fig. 4G). Nevertheless, in Mn starved medium, *smf12* mutant formed smooth colonies in contrast to the WT or the complemented strains that differentiated marked invasive filaments (Fig. 4G). This observation might explain the reduced virulence of *smf12* in the different tested infection models and supports the role of Mn homeostasis in promoting morphogenetic switch in *C. albicans*.

### Mn homeostasis modulates sensitivity to antifungals

**Fig 5:**
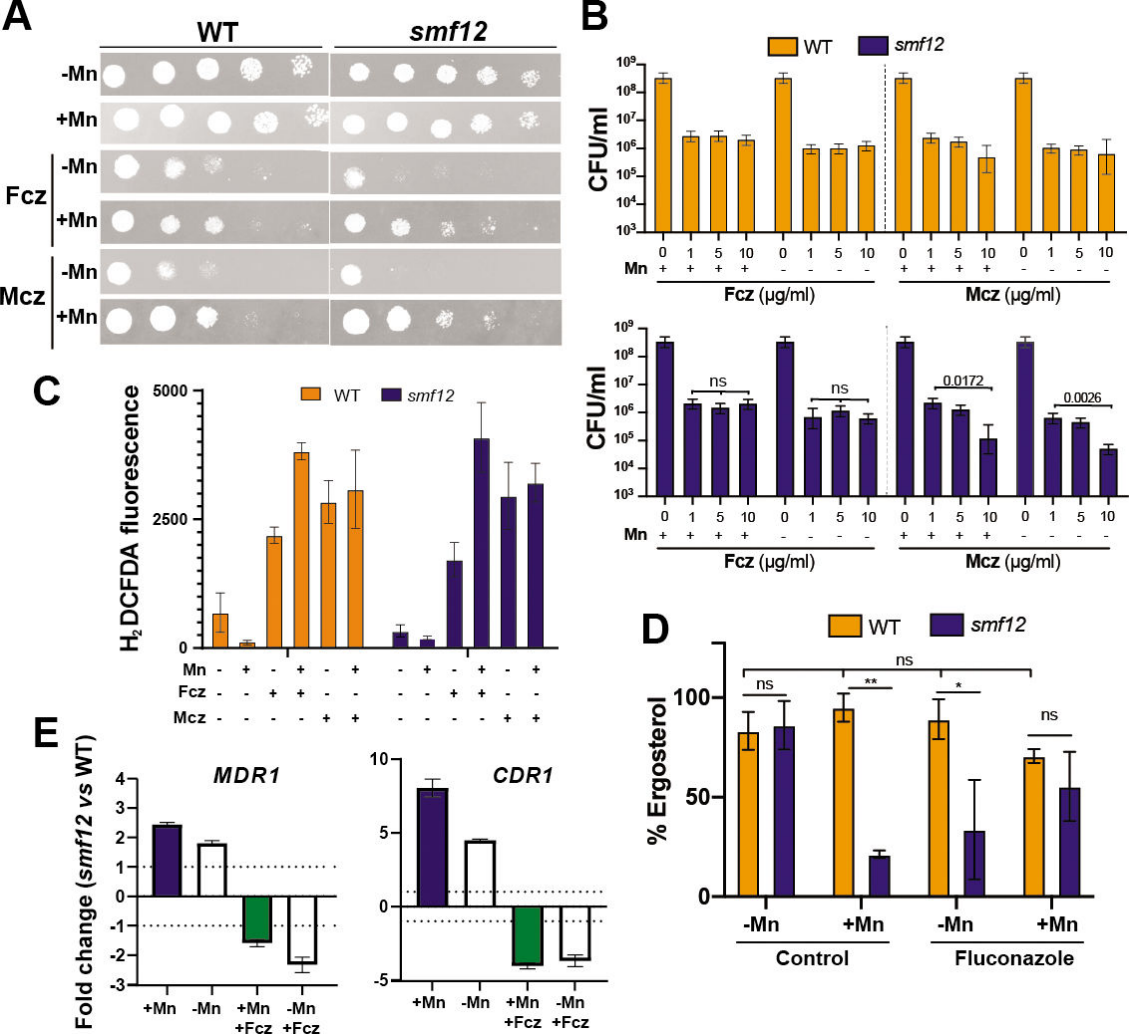
Mn homeostasis modulates antifungal sensitivity. (**A**) Growth assessment of WT and *smf12* mutant cells in the presence of fluconazole (Fcz; 1 µg/mL) and miconazole (Mcz; 0.5 µg/mL). *C. albicans* WT (SN148-CIp20) and *smf12* strains were serially diluted, spotted on SC-Mn and SC + Mn, and incubated for 2 days at 30°C. (**B**) Evaluation of the impact of Mn availability on WT (SN148-CIp20) and *smf12* mutant by CFU counts. Quantification of intracellular reactive oxygen species (**C**) and ergosterol levels (**D**) in WT (SN148-CIp20) and *smf12* cells. ** *P*-value < 0.001 and * *P*-value < 0.05; ns: nonsignificant. (**E**) Evaluation of transcript levels of *MDR1* and *CDR1* by qPCR. Transcript levels were assessed under Mn limitation and sufficiency in response to fluconazole, and fold changes were calculated using the comparative ΔCt method. Data were normalized using Ct values obtained from actin in each condition.

The impact of Mn homeostasis on antifungal stress was tested in both WT and *smf12* cells, growing in either Mn limitation or sufficiency. Our data show that sensitivity to fluconazole and miconazole in WT cells was moderately increased in Mn-starved medium as compared to Mn-replete condition (Fig. 5A). Furthermore, *smf12* was hypersensitive to the two tested azoles as compared to WT specifically in Mn-restricted condition (Fig. 5A). Assessing the number of colony forming unit (CFU) confirmed a decrease in cell viability for *smf12* as compared to the WT especially for miconazole treatment and under Mn limitation (Fig. 5B). Thus, in addition to UPR stress, Mn abundance modulates *C. albicans* tolerance to azole antifungals.

To underline the mechanism leading to antifungal sensitivity of either WT or *smf12* mutant under Mn limitation, we first tested whether reactive oxygen species (ROS) as a mediator of azoles antifungal activity was exacerbated (45). As Mn is a critical cofactor of *C. albicans* superoxide dismutases (28, 46), we hypothesized that antifungal sensitivity might be a consequence of impairment of the ROS-neutralizing capacity of *C. albicans* cells. Our data show that neither Mn depletion nor *smf12* mutation has a significant impact on ROS levels in either antifungal-treated or control cells (Fig. 5C). Second, as the cellular content of ergosterol is associated with azole sensitivity (47), we tested the impact of Mn availability and *smf12* mutation on the abundance of this fungal sterol. In WT cells, no significant change in ergosterol levels was seen in both fluconazole-treated and nontreated cells under either Mn scarcity or sufficiency (Fig. 5D). However, for *smf12* mutant*,* ergosterol amounts dropped significantly in cells growing in SC + Mn medium as compared to SC-Mn. This trend was inverted when *smf12* cells were challenged by fluconazole where ergosterol amount was reduced by approximately threefold under Mn starvation as compared to Mn replete condition (Fig. 5D). Thus, the reduced amount of ergosterol in *smf12* might explain the sensitivity of this mutant to azole antifungals. Lastly, we assessed the contribution of the two drug efflux pumps Cdr1 and Mdr1, which are key determinants of azole clinical resistance (48), to the Mn-modulated antifungal sensitivity by assessing their transcript levels using qPCR. Both *MDR1* and *CDR1* were significantly downregulated in *smf12* mutant in *C. albicans* challenged with fluconazole (Fig. 5E). Taken together, Mn modulation of antifungal sensitivity might operate through the regulation of antifungal efflux and the maintenance of ergosterol homeostasis.

### Global transcriptional response *of C. albicans* to Mn excess

**Fig 6:**
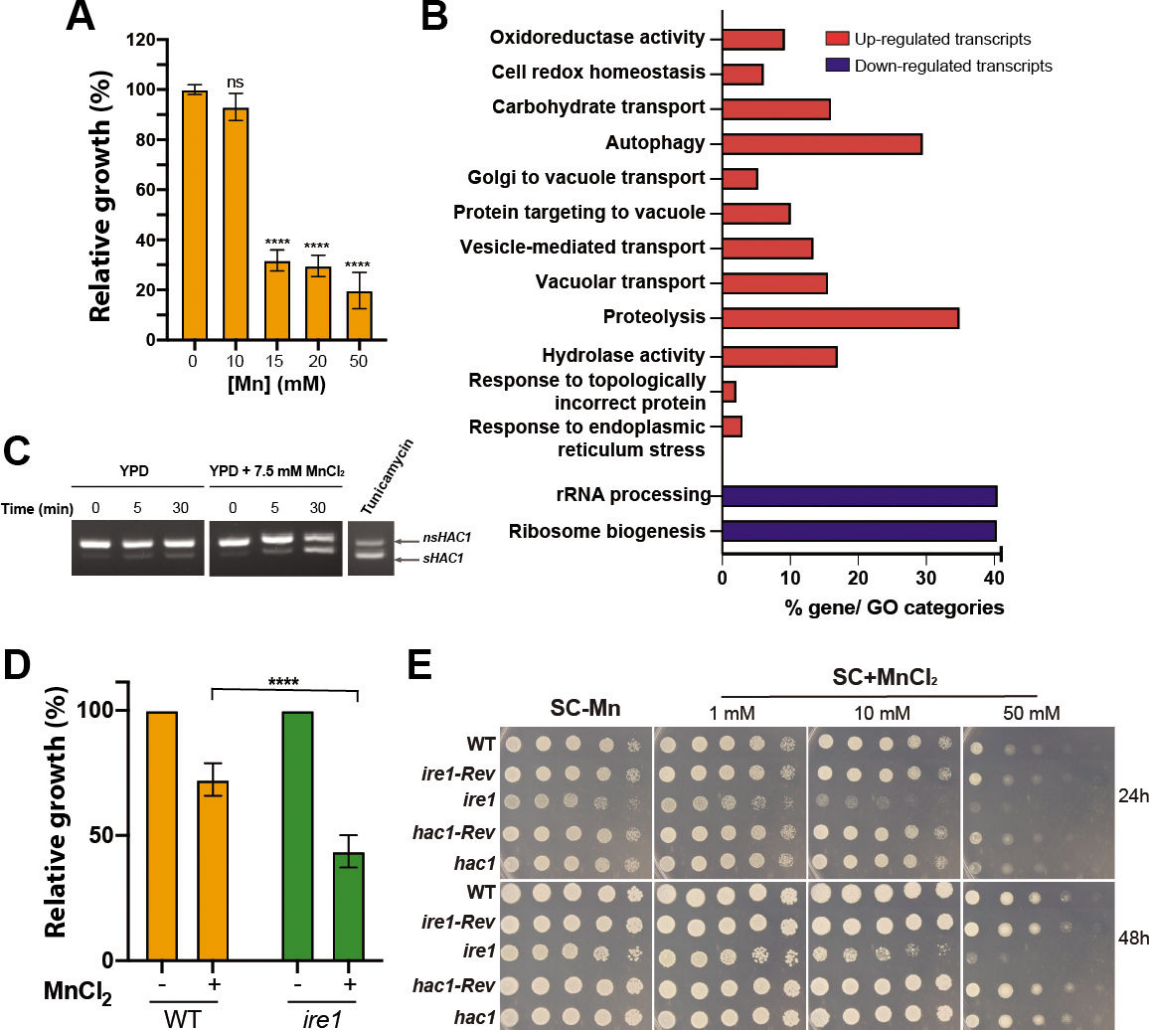
Impact of Mn excess on *C. albicans.* (**A**) Effect of increased Mn levels on *C. albicans* growth. SC5314 was grown in YPD supplemented with different concentrations of MnCl_2_ for 13 h at 30°C. Growth was normalized to the nontreated condition. (**B**) Transcriptional profiling of *C. albicans* cells under Mn excess. Overrepresented GO functional categories of differentially modulated transcripts are shown. (**C**) Mn toxicity induces *HAC1* mRNA splicing. WT cells were grown in YPD supplemented with 7.5 mM of MnCl_2_, and *HAC1* splicing was assessed using RT-PCR. Tunicamycin was used as a positive control. *nsHAC1*, nonspliced *HAC1; sHAC1*, spliced *HAC1*. Effect of Mn excess on *ire1* and *hac1* growth in both liquid (**D**) and solid medium (**E**). All strains were grown in liquid YPD with 7.5 mM of MnCl_2_ for 13 h at 30°C (**D**) or in YPD-agar medium with the indicated Mn concentrations (**E**). *ire1-Rev* and *hac1-Rev* are the *ire1* and *hac1* complemented strains, respectively. Statistics are ANOVA test with **** *P*-value < 0.00001; ns: nonsignificant.

While increasing Cu levels is thought to be an *in vivo* defense strategy to limit fungal infections by the host, nothing is known regarding the contribution of Mn to such a mechanism (49). A recent study uncovered a significant increase of Mn in response to systemic infection by *C. albicans* or under colitis (50). However, the relevance of such a phenomenon as an antifungal defense mechanism remains unexplored. We undertook RNA-seq profiling of *C. albicans* cells exposed to Mn excess to understand how fungal cells cope with such stress. The inhibitory effect of Mn excess on *C. albicans* growth was detected at 10 mM leading to 10% inhibition while 15 mM Mn caused 70% growth reduction (Fig. 6A). To capture the cellular processes impacted by Mn excess, we used RNA-seq profiling of cells exposed to 7.5 mM MnCl_2_, a concentration that led to 10% growth inhibition. GO enrichment analysis of upregulated transcripts uncovers a transcriptional signature of ER stress with enrichment of many biological functions including proteolysis, vesicle-mediated transport, protein folding, and response to oxidative stress which are *bona fide* hallmarks of UPR (Fig. 6B; Table S1). Transcripts related to ribosome biogenesis and ribosomal RNA processing were repressed. UPR activation by Mn excess was supported by Hac1 splicing and the requirement of Ire1 to tolerate higher concentrations of Mn (Fig. 6C through E). This finding implies that both Mn starvation and excess in *C. albicans* generate ER stress which promotes the activation of UPR signaling. Intriguingly, the inactivation of *HAC1* did not lead to sensitivity to Mn excess. This suggests that *Hac1* might act independently of Ire1 or that *hac1* defect is being buffered by a compensatory mechanism yet to be identified.

### Conclusion

Overall, the current study presents a comprehensive transcriptional portrait of biological functions that are modulated by Mn abundance in *C. albicans* cells. Some of these functions have been previously shown to be essential for host infection underscoring the value of targeting fungal Mn homeostasis for potential antifungal therapeutics development. This was also supported by our finding showing that inactivation of the Mn transporter *SMF12* led to attenuated virulence in different infection models. Furthermore, the transcriptional pattern of Mn starvation was similar to that expressed in different contexts of *C. albicans*-host interaction emphasizing that Mn scarcity is a situation encountered during infection. Importantly, we found that intracellular Mn levels influence the abundance of the essential metals Fe and Zn which emphasizes the complex crosstalk between metal ions. This could be explained by the fact that these metals might share common transporters as was reported for NRAMP transporters and their dual specificity toward Mn and Fe (51, 52). Alternatively, this might be related to the fact that Fe and Zn assimilation are mediated by proteins that are glycosylated in the secretory pathway, a process that relies on Mn-dependent glycosyltransferase (53–55). Overall, this work provides fertile areas for future studies to examine the link between Mn metabolism and essential functions that modulate fungal virulence and ﬁtness inside the host.

## MATERIALS AND METHODS

### Strains and growth conditions

The fungal strains used in this study are listed in Table S3. *C. albicans* clinical strain SC5314 (56) and its derivatives were routinely maintained at 30°C on SC [1.7% yeast nitrogen base (YNB), 0.5% ammonium sulfate, 2% dextrose, 0.2% amino acid, and 50 µg/mL uridine] or YPD media (Yeast extract–Peptone–Dextrose; 2% Bacto peptone, 1% yeast extract, 2% dextrose, and 50 µg/mL uridine). *smf12* deletion mutant (*smf12:URA3*/*smf12:HIS1*) was constructed from SN148 strain (57) by replacing the entire open reading frame (ORF) with a PCR-disruption cassette generated from pFA plasmids (58). A wild-type control strain (SN148-CIp20) was created by reintroducing *URA3* and *HIS1* in SN148 using the integrative Cip20 plasmid (59). For the complementation of *smf12* mutant, PCR primers were designed to amplify 1 kb upstream of *SMF12* in addition to the complete *SMF12* ORF. The resulting PCR products were cloned in the pDUP3 plasmid (60). The resulting pDUP3-*SMF12* construct was digested by *SfI1* and integrated into the *NEUT5L* genomic site of the *smf12* strain as previously described (60) using a lithium acetate transformation procedure (61). Transformants were selected on YPD plates supplemented with 200 µg/mL nourseothricin, and correct integration was verified by PCR. Primers used for *SMF12* cloning in pDUP3 plasmid and for the diagnosis of integration are listed in Table S3. Effect of Mn abundance on fungal growth was performed as follows: fungal inocula were prepared from an overnight culture grown in SC-Mn medium (Formidium) at 30°C and diluted to a starting OD_600_ of 0.1 in either SC without Mn (SC-Mn) prepared using YNB without Mn (Formidium) or supplemented with 1 mM of MnCl_2_ (SC + Mn). Cultures were exponentially grown for 13 h at 30°C under agitation. Relative growth was assessed using the growth on the SC medium as a control condition. The effect of Mn excess was tested similarly using a YPD medium with different concentrations of MnCl_2_, and growth was normalized to the nontreated condition. The filamentation assay was performed in spider medium (62). Exponentially growing *C. albicans* cells were seeded on a Spider-agar plate and incubated for 3 days at 37°C. All chemicals used in this study were provided by Sigma-Aldrich (St. Louis, MO, United States). Miconazole (1 mg/mL) and fluconazole (10 mg/mL) stock solutions were prepared using dimethyl sulfoxide. Working stock solutions of Mn (II) Chloride (MnCl_2_; 1 M) were prepared using Chelex resin-treated MilliQ water. For growth inhibition assays in liquid SC-Mn and SC + Mn media, overnight cultures of *C. albicans* were resuspended in fresh SC-Mn medium at an OD_600_ of 0.1 and added to a flat-bottom 96-well plate in a total volume of 200 µL per well along with the compounds being tested. For each experiment, a compound-free positive growth control and a cell-free negative control were included. Growth assay curves were performed in triplicate in 96-well plates using a Sunrise plate-reader (Tecan) at 30°C under constant agitation. The CFU assay was performed as follows: *C. albicans* cells were grown in the presence of 1, 5, or 10 µg/mL Fluconazole or Miconazole during 48 h at 30°C in 96-well plates using the Sunrise plate-reader (Tecan) under constant agitation. Plates were then centrifuged, washed with PBS, and spread on YPD plates at different dilutions. CFU was assessed after 24 h growth at 30°C.

### Inductively coupled plasma-mass spectrometry

The total amounts of cell-associated Mn were quantified from cultures that were grown exponentially to an OD_600_ of 0.5 on SC-Mn and SC + Mn. All vessels of yeast culture were washed with 10% nitric acid. Cells were centrifuged, washed with ice-cold metal-free PBS pH 7.4, and dried overnight at 65°C. Dried cells were weighed and digested in a mix of 1 mL NHO_3_ 70% and 2 mL H_2_O_2_ 30% and heated for 45 min at 95°C in a dry bath. Digested samples were diluted with 1% NHO_3_. For Mn quantification in SC and SC-Mn media, a total of 100 mg of YNB or YNB-Mn powders were digested as described for cell pellets. Elemental composition was analyzed by Thermo Scientific iCAP Q ICP-MS instrument (Laser-ablation ICP-MS Facility, Department of Earth and Planetary Sciences, McGill University). Metal concentrations were calculated from the standard curve of Mn (1–800 ppb Mn) and normalized to the pellet weight of each sample.

### Expression analysis by RNA-seq and quantitative PCR

Overnight cultures of SC5314 strain were diluted to an OD_600_ of 0.1 in 60 mL of fresh SC-Mn medium and grown at 30°C under agitation (200 rpm) to early logarithmic phase (OD_600_ = 0.4). Cultures were then either left untreated or supplemented with 1 mM MnCl_2_ and incubated at 30°C for 5 and 90 min. For RNA-seq profiling of Mn excess, overnight cultures of WT cells were diluted to an OD_600_ of 0.1 in fresh 100 mL YPD and incubated with shaking at 30°C to an OD_600_ of 0.8 and split into 50 mL cultures. MnCl_2_ was added to the experimental culture to a final concentration of 7.5 mM, while an equal volume of sterile water was added to the control culture and incubated for 30 min. Cells were harvested by centrifugation and were flash-frozen and stored at −80°C. For each condition, a total of two biological replicates were considered for RNA-seq analysis. Total RNA was extracted using an RNAeasy purification kit (Qiagen) and glass bead lysis in a Biospec Mini 24 bead-beater as previously described (63). RNA integrity was assessed using the Agilent 4200 Tape Station System prior to cDNA library preparation. The NEBNext Ultra II RNA Library Prep Kit for Illumina was used to construct the RNA-seq library following the manufacturer’s instructions. A 2 × 100 paired-end sequencing of cDNAs was performed using an Illumina NovaSeq 6000 sequencing system. The GSEA preranked tool (http://www.broadinstitute.org/gsea/) was used to determine the statistical significance of correlations between the *C. albicans* Mn-sensitive transcriptomes with GO biological process terms and different omics data sets as described in references (39, 64). Differentially expressed transcripts in Table S1 were identified using Welch’s *t* test with a false-discovery rate of 5% and a 1.5-fold enrichment cut-off. GO analysis was performed using GO Term Finder of the Candida Genome Database (65). All RNA-seq data are available at the GEO database (https://www.ncbi.nlm.nih.gov/geo/) with the accession number GSE245114.

For quantitative PCR (qPCR) experiments of *MDR1* and *CDR1* transcripts, a total of three biological and three assay replicates were performed. Cells were grown exponentially to OD_600_ = 0.5 and treated with fluconazole (1 µg/mL) in either SC-Mn or SC + Mn for 90 min. RNAs were extracted for the RNA-seq experiment. cDNA was synthesized from 1 µg of total RNA using the High-Capacity cDNA Reverse Transcription kit (Applied Biosystems). The mixture was incubated at 25°C for 10 min, 37°C for 120 min, and 85°C for 5 min. RNAse H (NEB) was added to remove RNA, and samples were incubated at 37°C for 20 min. qPCR was performed using a StepOnePlus Instrument (Applied Biosystems) for 40 amplification cycles with the PowerUp SYBR Green master mix (Applied Biosystems). The reactions were incubated at 95°C for 10 min and cycled 40 times at 95°C, 15 s; 60°C, 1 min. Fold-enrichment of each tested transcript was estimated using the comparative ΔΔCt method. To evaluate the gene expression level, the results were normalized using Ct values obtained from Actin (*ACT1*, C1_13,700W_A). Primer sequences used for this analysis are summarized in Table S3.

### HAC1 mRNA splicing assay

The *HAC1* splicing was assessed using RT-PCR as previously described (66). RNAs were extracted from *C. albicans* cells challenged with tunicamycin (10 µg/mL), used as a positive control, or in the absence or the presence of Mn (1 mM MnCl_2_ for Mn repletion and 7.5 mM for Mn excess experiments) as described in the RNA-seq experiment. cDNAs were obtained using Superscript II reverse transcriptase (Applied BioSystems) as recommended by the supplier. The obtained cDNA was used as a template to amplify spliced and unspliced *HAC1* cDNAs using the primer pair described in reference (66). The PCR products were resolved on 4% agarose gel. PCR band intensities were quantified with ImageJ (67).

### *Galleria* virulence assay

Larvae of *G. mellonella* (Elevages Lisard, Canada) in the instar larval stage of development were used. Overnight cultures of *C. albicans* strains were washed twice and diluted in 20 µL PBS to obtain a quantity of 5 × 10^5^ cells in 20 µL for injection. *G. mellonella* larvae weighing 180 ± 10 mg were injected between the third pair of prothoracic legs. Infected larvae were incubated at 37°C. Two replicates, each consisting of 20 larvae, were carried out with survival rates measured daily for 5 days. Death was determined based on the lack of response to touch and the inability to right themselves. Kaplan-Meier survival curves were created and compared with the log-rank test (GraphPad Prism 5).

### HT-29 and J774A.1 damage assay

Damage to the human colon epithelial cell line HT-29 (ATCC; HTB-38) and the murine J774A.1 (ATCC TIB-67) macrophages was assessed using a lactate dehydrogenase (LDH) cytotoxicity detection kit*PLUS* (Roche), which measures the release of the LDH enzyme in the growth medium. HT-29 and J774A.1 cells were grown in 96-well plates as monolayers in McCoy’s medium and Dulbecco’s Modified Eagle’s Medium, respectively, supplemented with 10% fetal bovine serum (FBS) at 2 × 10^4^ cells per well and incubated at 37°C with 5% CO_2_ overnight. Cells were then infected with *C. albicans* cells precultured in the presence (SC + Mn) or the absence of Mn (SC-Mn), at MOI (multiplicity of infection) cell:yeast of 1:2 for 24 h at 37°C with 5% CO_2_. Following incubation, 100 µL of supernatant was removed from each experimental well, and LDH activity in this supernatant was determined by measuring the absorbance at 490 nm following the manufacturer’s instructions. Cytotoxicity was calculated as follows: % cytotoxicity = [experimental value − low control (untreated cells)] / [high control (lysis buffer) − low control] × 100.

### Glycosylation assay

*C. albicans* cells were grown in SC-Mn and SC + Mn for the RNA-seq experiments. Cells were harvested by centrifugation and lysed by bead beating in IP150 buffer (50 mM Tris-HCl at pH 7.4, 150 mM NaCl, 2 mM MgCl_2_, 1% Nonidet P-40, and 5% glycerol) supplemented with Complete Mini protease inhibitor mixture tablet (Roche Applied Science) and 10 mM phenylmethylsulfonyl fluoride. The lysates were then cleared by centrifugation, and protein concentration was estimated using the Bradford assay. A total of 80 µg of proteins were heated for 5 min at 95°C in the 2X Laemmli buffer and loaded in an Acrylamide gel. Gels have been stained with silver to observe the total protein profile or with Pierce Glycoprotein Staining Kit (Thermo Fisher) to detect glycoprotein sugar moieties according to the manufacturer’s instructions.

### ROS quantification

Intracellular ROS was measured using the oxidative stress indicator H_2_DCFA-DA dye (50 µg/mL final concentration; Invitrogen, ThermoFisher Scientific) to quantify the level of reactive oxygen species. Cells were grown as described in the qPCR experiment.

### Ergosterol quantification

Ergosterol quantification was performed as previously described (68). Cells were grown exponentially to OD_600_ = 0.5 and treated with fluconazole (1 µg/mL) either SC-Mn or SC + Mn for 16 h. Cells were then washed, weighted, resuspended in ethanolic potassium hydroxide at 25%, and incubated for 2 h in a 95°C water bath. After cooling, 3 mL n-heptane (Heptane, 99%) and 1 mL H_2_O were added. Fluorescence of the supernatants was measured at 282 and 230 nm.

## Data Availability

The original contributions presented in the study are included in the Supplementary Material. RNA-seq data have been submitted to the GEO database under accession number GSE245114. Further inquiries can be directed to the corresponding author.

## SUPPLEMENTAL MATERIAL

Table S1 msphere.00804-23-s0001.xlsx Transcripts differentially expressed in *C. albicans* WT under Mn starvation and excess.

Table S2 msphere.00804-23-s0002.xlsx GSEA of the *C. albicans* Mn-modulated transcriptome.

Table S3 msphere.00804-23-s0003.xlsx Strains and primers used in this study.

ASM does not own the copyrights to Supplemental Material that may be linked to, or accessed through, an article. The authors have granted ASM a non-exclusive, world-wide license to publish the Supplemental Material files. Please contact the corresponding author directly for reuse.
